# Evaluating the suitability of close‐kin mark‐recapture as a demographic modelling tool for a critically endangered elasmobranch population

**DOI:** 10.1111/eva.13474

**Published:** 2022-09-03

**Authors:** Aurélien Delaval, Victoria Bendall, Stuart J. Hetherington, Hans J. Skaug, Michelle Frost, Catherine S. Jones, Leslie R. Noble

**Affiliations:** ^1^ Faculty of Biosciences and Aquaculture Nord University Bodø Norway; ^2^ Centre for Environment Fisheries and Aquaculture Science (CEFAS) Lowestoft UK; ^3^ Department of Mathematics University of Bergen Bergen Norway; ^4^ School of Biological Sciences University of Aberdeen Aberdeen UK

**Keywords:** abundance estimation, blue skate, close‐kin mark‐recapture, conservation, elasmobranch, fisheries

## Abstract

Estimating the demographic parameters of contemporary populations is essential to the success of elasmobranch conservation programmes, and to understanding their recent evolutionary history. For benthic elasmobranchs such as skates, traditional fisheries‐independent approaches are often unsuitable as the data may be subject to various sources of bias, whilst low recapture rates can render mark‐recapture programmes ineffectual. Close‐kin mark‐recapture (CKMR), a novel demographic modelling approach based on the genetic identification of close relatives within a sample, represents a promising alternative approach as it does not require physical recaptures. We evaluated the suitability of CKMR as a demographic modelling tool for the critically endangered blue skate (*Dipturus batis*) in the Celtic Sea using samples collected during fisheries‐dependent trammel‐net surveys that ran from 2011 to 2017. We identified three full‐sibling and 16 half‐sibling pairs among 662 skates, which were genotyped across 6291 genome‐wide single nucleotide polymorphisms, 15 of which were cross‐cohort half‐sibling pairs that were included in a CKMR model. Despite limitations owing to a lack of validated life‐history trait parameters for the species, we produced the first estimates of adult breeding abundance, population growth rate, and annual adult survival rate for *D. batis* in the Celtic Sea. The results were compared to estimates of genetic diversity, effective population size (*N*_*e*_), and to catch per unit effort estimates from the trammel‐net survey. Although each method was characterized by wide uncertainty bounds, together they suggested a stable population size across the time‐series. Recommendations for the implementation of CKMR as a conservation tool for data‐limited elasmobranchs are discussed. In addition, the spatio‐temporal distribution of the 19 sibling pairs revealed a pattern of site fidelity in *D. batis*, and supported field observations suggesting an area of critical habitat that could qualify for protection might occur near the Isles of Scilly.

## 1 INTRODUCTION

Estimating contemporary population demographic parameters such as census size (*N*_*c*_) is fundamental to understanding a species' recent evolutionary history, and is essential for effective conservation of endangered species. The status of marine fish populations is often assessed by estimating their relative abundance based on catch per unit effort (CPUE), obtained from data on commercial landings and/or fisheries‐independent surveys. However, CPUE estimates can be biased by misreporting of catches and variations in catchability of the animals with different sampling gears (Maunder & Piner, 2015). Data limitations are particularly pertinent to elasmobranchs, assessments of which are often hindered by taxonomic confusion (Iglésias et al., 2010) and insufficient knowledge of their biology (ICES, 2020; IUCN, 2015). These data limitations represent a major conservation concern; many elasmobranchs have suffered population declines and local extinctions as a result of fishing pressure over the past century, and many are still caught as bycatch despite landing bans (ICES, 2020; Simpson & Sims, 2016).

Alternatively, mark‐recapture approaches can be used to estimate absolute abundance (Cormack, 1964; Jolly, 1965; Seber, 1965), with the added benefit that they can reveal patterns of animal movement and habitat use. As a result, mark‐recapture has been a popular approach to study elasmobranch species (Biais et al., 2017; Corrigan et al., 2018; Neat et al., 2015). However, applications to benthic elasmobranchs such as batoids have generally suffered from low animal recapture and tag recovery rates (Bendall et al., 2018; Bird et al., 2020). For elasmobranch populations facing methodological constraints such as these, novel approaches are urgently needed to estimate recent population trends and inform conservation decisions.

Rapid advances in molecular genetic approaches in the last few decades have provided novel means of assessing population trends. Close‐kin mark‐recapture (CKMR) has recently emerged as a method of estimating demographic parameters such as absolute abundance, population growth rates, and survival rates (Bravington, Skaug, et al., 2016). CKMR builds on traditional mark‐recapture approaches by making use of genetic data obtained from small biopsies (e.g. muscle or fin clips) to identify closely related individuals within a population; here, genotypes can be considered as ‘tags’ and individuals' relatives in a sample can be considered as ‘recaptures’ based on the principles of Mendelian inheritance. The principles of mark‐recapture are conserved in CKMR, in that a higher proportion of recaptures reflects a smaller population size. Since the method requires only a single capture of individuals, circumventing the need for physical recaptures and the additional stress this would inflict on the animals, it represents a promising approach requiring less time at sea to assess a critically endangered elasmobranch population.

The idea behind CKMR is by no means new. Skaug (2001) and Nielsen et al. (2001) initially proposed that individual genotypes can function as genetic tags from which abundance can be estimated. However, genotyping and identifying close kin among large populations has only become feasible following recent advances in sequencing technologies, such as next‐generation sequencing (NGS), which have enabled the genotyping of large numbers of samples across the many loci (several thousand) required to accurately identify close kin. Thus far, CKMR has been successfully applied to populations of only a handful of species, including southern bluefin tuna *Thunnus maccoyii* (Bravington, Grewe, et al., 2016), a few salmonids (Prystupa et al., 2021; Ruzzante et al., 2019; Wacker et al., 2021), white shark *Carcharodon carcharias* (Hillary et al., 2018), grey nurse shark *Carcharias taurus* (Bradford et al., 2018) and thornback ray *Raja clavata* (Trenkel et al., 2022).

In addition to facilitating a novel demographic modelling approach, the identification of close kin can provide insights on elasmobranch behaviour and habitat use, which can reveal areas that may qualify for additional protective measures. For example, the identification of cross‐cohort siblings of speartooth sharks *Glyphis glyphis* across three Australian river systems revealed patterns of adult movement and breeding behaviour (Feutry et al., 2017), while the high degree of genetic relatedness within aggregations of basking sharks *Cetorhinus maximus* in the North‐East Atlantic revealed kin‐associated behaviour and identified potentially important migratory corridors for this species (Lieber et al., 2020).

The blue skate *Dipturus batis* (Linnaeus, 1758) is a large‐bodied rajid with a patchy distribution across the North‐East Atlantic Ocean, occurring in higher densities in the Celtic Sea and Rockall (Frost et al., 2020). *D. batis* was only recently differentiated from its larger congener, the flapper skate *D. intermedius*, following recent morphological and genetic investigations (Griffiths et al., 2010; Iglésias et al., 2010). Much of the presumed knowledge on both species is being re‐assessed and still lacks detail, and as a result, management will continue on the basis of a single common skate complex (*D. batis* complex) until species‐specific assessments improve (ICES, 2020). At present, both species are classed as Critically Endangered by the IUCN (Dulvy et al., 2006). Current management implies a total landing ban on common skate in EU waters since 2009, though post‐ban landings and bycatch are still reported (ICES, 2020; Simpson & Sims, 2016).

Anecdotal reports indicate high rates of common skate bycatch in the Celtic Sea, an important conservation and socio‐economic concern in the context of a multi‐species fishery. Initial surveys identified that common skate bycatch in the Celtic Sea is dominated by *D. batis* (Bendall et al., 2018). In an effort to evaluate current management regimes, longitudinal fisheries‐dependent surveys have been initiated in collaboration with British (Bendall et al., 2018) and French (Barreau et al., 2016) fishing fleets in order to develop demographic models and identify biologically important sites for the species. Initial results from these surveys suggest the occurrence of juveniles and adults in the area, displaying a high degree of site fidelity, while population genomic analysis has identified the occurrence of siblings in the area and limited gene flow with offshore populations (Delaval et al., 2022). Altogether, these findings suggest that the Celtic Sea may host important reproductive and/or nursery sites for *D. batis*.

The Celtic Sea *D. batis* population represents a unique opportunity to test the suitability of CKMR as a demographic modelling tool for a data‐deficient benthic elasmobranch; the apparently site‐attached nature of *D. batis* in the Celtic Sea, the occurrence of close kin and the longitudinal samples available from standardized surveys make it an ideal candidate for CKMR. In this study, we applied a genome‐wide genotyping approach (DArTseq™, Kilian et al., 2012) on samples collected during fishery‐dependent surveys in the Celtic Sea between 2011 and 2017 to identify half‐siblings and generate the first estimates of the number of breeding adults (*N*), adult population growth rate and adult survival rate for the population using CKMR. To evaluate the suitability of CKMR as applied to this population, these estimates were compared to molecular estimates of *N*_*e*_ (the evolutionary analogue of *N*, see Waples et al., 2018) and genetic diversity, and to relative abundance estimates (CPUE) obtained from the survey. In addition, the spatio‐temporal distribution of close kin would allow us to evaluate skate movements and identify potential areas of biological interest that may qualify for additional protection.

## 2 METHODS

### 2.1 Sample collection and genotyping

**FIGURE 1:**
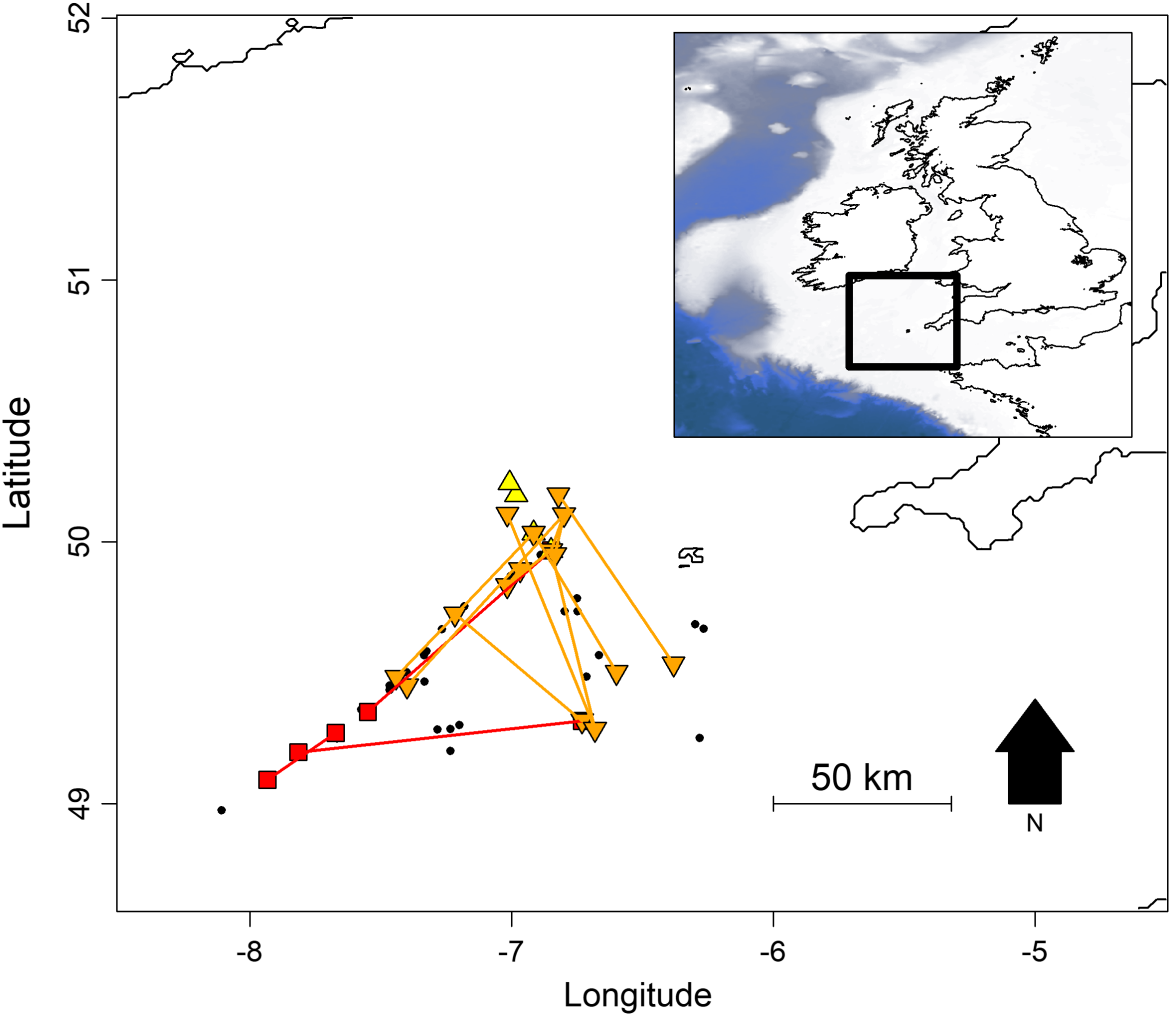
Sampling locations of all *Dipturus batis* in this study (black points), full‐siblings (red squares), and half‐sibling pairs (orange and yellow triangles). Straight lines are drawn between full‐sibling (red) and half‐sibling (orange) pair capture locations. Half‐siblings captured in the same haul are indicated by yellow triangles. Latitude and longitude are in decimal degrees.

Fishery‐dependent common skate surveys were performed in the Celtic Sea in collaboration with the fishing industry in 2011, and from 2014 to 2017. The surveys, which ran in early autumn (August–October), sampled using fixed trammel nets along a transect of stations running 12‐80 NM to the south and west of Newlyn, Cornwall, UK (Figure 1). Additional exploratory stations outside the transect area were also surveyed to assess the extent of *D. batis*' distribution. Further details on the sampling protocol are described in Bendall et al. (2018). Across survey years, biopsies (fin or muscle clips) were taken from 1140 individuals and stored in 96% ethanol or RNAlater®. Skates were also sexed and measured (total length, cm). A pilot study was performed to determine the power of relationship inference using DArTseq™ genotyping, and the probability of obtaining parent‐offspring or half‐sibling pairs among the samples (Appendix S1). The results revealed that parent‐offspring pairs were unlikely to be found, as samples mostly comprised young adults, whereas half‐sibling pairs (HSPs) could be identified with high levels of precision. Based on these results, we opted for a HSP CKMR approach.

Due to cost considerations, we were unable to genotype all samples. Of those available, 683 were selected for genotyping, of which 387 had already been genotyped by Delaval et al. (2022) using the same method. Samples primarily consisted of juveniles and young adults, but spanned as wide a size range as possible to maximize the number of cohorts included in the model. Genomic DNA was extracted using a DNeasy® Blood & Tissue kit (Qiagen), quantified on a Qubit fluorometer (Thermo Fisher Scientific) and adjusted to 10–60 ng/μl prior to sequencing. To assess the suitability of the DNA for restriction enzyme digestion, we performed a mock sample digest in CutSmart® Buffer (New England Biolabs) for 2 h at 37°C, and resolved all samples on 0.8% TAE electrophoresis gels. In addition, 33 samples were visualized on a Genomic DNA ScreenTape® for a more detailed visualization of DNA quality. DNA was then sent to Diversity Arrays Technology (DArT) Pty. Ltd. for genotyping using DArTseq™ technology.

Genotyping was performed following standard protocols as described in Kilian et al. (2012). DArTseq™ combines complexity reduction methods and NGS platforms, and is optimized for each organism. Based on tests of several enzyme combinations for complexity reduction, DArT Pty. Ltd. applied the restriction enzyme combination *PstI* and *SphI* on the samples, which were sequenced (single read) on an Illumina® HiSeq® 2500, generating approximately 1.5 million sequences per individual. Sequences were processed using proprietary DArT Pty. Ltd. analytical pipelines, generating data for 25,131 sequences of 69 bp length, each containing a single nucleotide polymorphism (SNP).

### 2.2 SNP filtering

Single nucleotide polymorphisms were filtered based on a call rate of 95%, and when duplicate loci were present, only that locus with the highest call rate was retained. After this step, the proportion of scored loci per sample was assessed; all samples had a sufficiently high score rate (≥90%) to be retained. Monomorphic loci and those with low minor allele frequencies (MAF < 0.05) were identified using *adegenet* (v 2.1.2, Jombart, 2008; Jombart & Ahmed, 2011), as implemented in R (v 3.6.2, R Core Team, 2019), and subsequently removed. Next, we tested for conformation of loci to Hardy–Weinberg proportions using the R package *pegas* (v 0.12, Paradis, 2010), performing an exact test based on Monte Carlo permutation of alleles (Guo & Thompson, 1992) with 1000 replicates. After applying the false discovery rate correction method of Benjamini and Hochberg (1995), loci were removed if they deviated significantly from Hardy–Weinberg equilibrium (at a significance threshold of α = 0.05). We then tested for linkage disequilibrium (LD) among loci using the R package *snpStats* (v 1.36.0, Clayton, 2020) and removed one locus from each pair of loci for which *R* ^2^ > 0.80. Because human error could lead to sampling an individual multiple times or to contamination during molecular laboratory work, we looked for duplicate samples based on a threshold of 629 mismatching loci (roughly 10% of remaining loci) using the R package *CKMRsim* (Anderson, https://doi.org/10.5281/zenodo.3519358). Where duplicates were found (i.e. >90% genetically identical), only the sample with the highest score rate was retained. Following these filtering steps, summarized in Table S1, the resulting dataset contained 662 individuals genotyped at 6291 loci (Table 1, Figure 1).

**TABLE 1:** Final panel of 662 *Dipturus batis* from the Celtic Sea incorporated into a close‐kin mark‐recapture model

Year sampled	Sample size (male, female)	Mean length in cm (range)	Mean estimated age (range)
2011	158 (69, 89)	122 (75, 148)	9 (3, 27)
2014	155 (84, 71)	121 (75, 147)	9 (3, 23)
2015	204 (109, 95)	115 (66, 146)	8 (2, 21)
2017	145 (66, 79)	113 (69, 142)	8 (2, 16)
Total	662 (328, 334)	118 (66, 148)	8 (2, 27)

### 2.3 Identification of kin‐pairs

Related individuals were identified using *CKMRsim*, which simulates related pairs of individuals based on observed allele frequencies using a Monte Carlo approach. *CKMRsim* calculates the false positive and false negative rates at different log‐likelihood thresholds for pairwise hypothesis tests involving different relationship categories (e.g. parent‐offspring PO, full‐sibling FS, half‐sibling HS, first‐cousin FC, and unrelated U). Due to the large number of pairwise comparisons in relationship testing (662 samples imply 218,791 pairwise tests), this approach enables the user to identify an appropriate log‐likelihood threshold, given the corresponding error rates, when performing the relationship tests. Details about the *CKMRsim* analysis are provided in Appendix S3. For the sake of comparison with other relatedness‐finding methods, we also searched for related pairs using *ML‐relate* (Kalinowski et al., 2006) and calculated pairwise relatedness (*r*) using the Wang estimator in the R package *related* (v. 1, Pew et al., 2015).

### 2.4 Close‐kin mark‐recapture

Close‐kin mark‐recapture builds upon traditional mark‐recapture (MR) abundance estimation by integrating information on the relatedness of individuals. In short, CKMR consists of determining the relationship (in this case as half‐siblings or not) of all pairs of individuals in a sample and comparing these to the prior probability of relatedness, given the life‐history parameters. Calculating these prior probabilities amounts to building a population dynamics model. The life‐history parameters, if known for the species, can include sex‐specific growth rates, age at maturity, and fecundity‐at‐age. This component of the model is flexible and can be adapted to the species in question by the addition or removal of life‐history parameters (Bravington, Skaug, et al., 2016). Models for data‐deficient species are by necessity simplified, requiring a number of assumptions to be made. After accounting for the observed related and unrelated pairs inferred from the genetic data, the population dynamics model then feeds into a demographic model from which initial population size and population growth rate can be estimated. In a HSP model, adult survival rates can also be estimated using information on the time interval between siblings' birth years, inferred from their estimated ages.

The HSP approach requires knowledge of the age of the individuals at the time of sampling. When sampling non‐lethally in the field, this implies estimating an individual's age based on its size. The data‐deficient nature of *D. batis* means little is known of its life‐history traits. Through a mark‐recapture experiment, Barreau et al. (2016) were able to use a sclerochronological approach using vertebral growth readings to propose an age‐at‐length model for *D. batis*, using the von Bertalanffy (1938) growth equation (Equation 1), which is commonly used to model fish size at age. We estimated the age (in years *t*) of each individual based on their length (in cm *L*) using this equation, rounding down to the year (Barreau et al., 2016).

(1) $$L_{t}=149\left[1-e^{-0.18\left(t+0.49\right)}\right]$$

We recognize that the parameters of this equation still require validation from further recaptures (Barreau et al., 2016), and that they lack defined uncertainty bounds that should ideally be included in a CKMR model. However, they offer the best method currently available to estimate age‐at‐length for *D. batis*. In order to assess the sensitivity of our CKMR model to erroneous age estimates, we repeated the CKMR analysis described below in a (less computationally demanding) maximum‐likelihood framework after re‐assigning ages to individuals according to their size (see Appendix S5). Owing to a lack of further available life‐history data, we opted for a sex‐ and age‐aggregated population dynamics model (Equation 2). That is, we assumed equal growth rates for both males and females, and constant adult survival rates over time. This approach is similar to that applied to other elasmobranchs to date (Bradford et al., 2018; Hillary et al., 2018), which also lacked precise life‐history parameters.

In our HSP population dynamics model (Equation 2), the probability that the relationship *K* between any two individuals *i* and *j* is half‐sibship (HSP), given their respective birth years (*b*) and that *j* was born after *i*, is dependent on the number of potential parents (i.e. number of breeding adults *N*) in the year of *j*'s birth and on the adult survival rate (φ)

(2) $$P\left[K_{\mathrm{ij}}=\mathrm{HSP}|b_{i},b_{j};b_{i}<b_{j}\right]=\frac{4}{N_{b_{j}}}\times \varphi^{\left(b_{j}-b_{i}\right)}$$

The rationale behind this formula, which is adapted from Hillary et al. (2018), is firstly that the probability that *i*'s mother ($M_{i}$, say) survives from $b_{i}$ to $b_{j}$ is $\varphi^{b_{j}-b_{i}}$, and secondly, the probability that *j* is the offspring of $M_{i}$ is $2/N_{b_{j}}$. The latter follows because there are $N_{b_{j}}/2$ candidate mothers at time $b_{j}$. Finally, the factor 2 comes from also taking the father into account.

The fact that *j* is $M_{i}$’s offspring with probability $2/N_{b_{j}}$ builds on the assumptions that the population is closed. Indeed, the closed population assumption may be valid, given that migration in and out of the Celtic Sea is apparently limited based on genetic (Delaval et al., 2022) and tagging (Bendall et al., 2018) data. Regarding the issue of differential probability of capture across space and time, the distribution of *D. batis* in the Celtic Sea is characterized by biological hotspots and spatially variable CPUE (Bendall et al., 2018). To minimize any potential bias, we selected samples randomly across survey stations and years.

In the demographic model (Equation 3), the number of adults in any given year *t* ($N_{t}$) is dependent on the number of adults in the initial model year (*t* = 0, i.e. the birth year of the first cohort in the sample set) and the annual population growth rate (r).

(3) $$\;N_{t}=N_{t=0}\times e^{\mathrm{rt}}\;$$

Our aim was to estimate initial adult breeding abundance ($N_{t=0}$), population growth rate (r), and annual adult survival rates (φ), and use this information to estimate abundance across the time series ($N_{t}$). We used a Bayesian approach, allowing us to set biologically meaningful constraints for each parameter and to visualize the credible intervals a‐posteriori. We implemented a Metropolis‐Hastings Markov‐Chain Monte Carlo (MCMC) model in R, whereby the posterior probability was calculated as follows:

(4) $$P\left(N_{t=0},r,\varphi |K_{\mathrm{ij}};i<j\right)\propto P\left(N_{t=0}\right)P\left(r\right)P\left(\varphi \right)\prod_{i<j}P\left(K_{\mathrm{ij}}|N_{t=0},r,\varphi \right)\;$$

where the proportionality symbol ∝ is used because we omit the normalization constant in Bayes' formula. Further, the term $P\left(N_{t=0}\right)P\left(r\right)P\left(\varphi \right)$ is a non‐informative prior on the parameters $N_{t=0}$, r and φ. Finally, $\prod_{i<j}P\left(K_{\mathrm{ij}}|N_{t=0},r,\varphi \right)$ is a product across all pairs $\left(i,j\right)$ of individuals in the data. For half‐siblings the probability $P\left(K_{\mathrm{ij}}=\text{HSP}|N_{t=0},r,\varphi \right)$ is given by Equation (2), while for unrelated individuals it is $1-P\left(K_{\mathrm{ij}}=\text{HSP}|N_{t=0},r,\varphi \right)$. Note that the parameter $N_{t=0}$ enters into Equation (2) through Equation (3). Because the set of pairwise relationships $K_{\mathrm{ij}}$ are not statistically independent (e.g. individuals *i* and *j* occur multiple times) unless a small portion of the population has been sampled, Equation (4) is not the exact posterior distribution, but corresponds instead to the pseudo‐likelihood of Bravington, Skaug, et al. (2016).

The Bayesian machinery requires priors to be assigned to all parameters, and in terms of r and φ, we used the priors to restrict the range of the parameters to ones that are biologically feasible (i.e. between −1 and 1 for r and between 0 and 1 for φ). We experimented with modelling parameters using short runs of 100,000 iterations, evaluating the performance of the models in Tracer (v 1.7.1, Rambaut et al., 2018) by assessing the distribution of parameter estimates ‘sampled’ by the Markov chain, and assessing the effective sample size for each parameter. The final MCMC was run for 1 million iterations, sampling every 100th iteration, and discarding the first 100,000 iterations as burn‐in. New variables for $N_{t=0}$, r and φ were proposed using a Metropolis‐Hastings sampler, and accepted based on the posterior acceptance ratio approach. Further details regarding the MCMC computation are provided in Appendix S4.

### 2.5 Genetic diversity and effective population size

Population‐ and locus‐wide summary statistics were obtained using GenAlEx (v 6.5, Peakall & Smouse, 2006, 2012). We estimated effective population sizes (*N*_*e*_) using the LD estimator (Hill, 1981; Waples, 2006; Waples & Do, 2010) in NeEstimator (v 2.1, Do et al., 2014). *N*_*e*_ is a theoretical estimator of population size that reflects the degree of genetic drift and, thereby, the evolutionary potential of wild populations; conservation thresholds of *N*_*e*_ have generally been set to 500 or 5000 individuals to mitigate the loss of genetic diversity and inbreeding depression, though these thresholds are debated (reviewed in Allendorf et al., 2013). The LD estimate assumed random mating, and we report the results when setting a critical value (i.e. MAF at which alleles should be excluded) of 0.05. Confidence intervals were obtained using the Jackknife‐over‐individuals method. *N*_*e*_ was calculated for the samples overall, and for each sampling year.

### 2.6 Catch per unit effort

In order to contextualize our estimates of population size and growth trends obtained from CKMR, we estimated relative total abundance (i.e. reflecting abundance of all individuals in the population, not just the breeders) by calculating CPUE, a metric that is more familiar to fisheries management.

We calculated annual CPUE rates from *D. batis* captured during fishery‐dependent common skate surveys performed in the Celtic Sea by CEFAS in collaboration with the fishing industry from 2014 to 2017, described in Bendall et al. (2018). All *D. batis* caught during the survey were counted and measured. Individual weights 𝑊_𝑇_ (g) were estimated from the parameters given by Silva et al. (2013):

(5) $$W_{T}=0.0038\times {L_{T}}^{3.1201}$$

Fishing effort was defined as kilometre‐hours (km h) of net soaked:

(6) $$\text{Unit effort}=\text{Length of}\;\mathrm{net}\;\left(\mathrm{km}\right)\times \text{Soak time}\;\left(\text{hours}\right)\;$$

Catch per unit effort was calculated for each station, as abundance (individuals km^−1^ h^−1^) and biomass (kg km^−1^ h^−1^), based on the number of individuals 𝑁_𝑇_ and summed weights *W*_*T*_ converted to kg respectively:

(7) $$\text{Abundance}=\frac{N_{T}}{\text{Unit effort}}$$

(8) $$\text{Biomass}=\frac{\sum W_{T}}{\text{Unit effort}}$$

For each year surveyed, mean abundance and biomass were calculated for (a) all stations fished, and (b) for prime stations that were fished each year (2014–2017, see Bendall et al., 2018). Where four stations were fished multiple times, the average was calculated for the four stations prior to averaging across the four stations.

## 3 RESULTS

### 3.1 Identification of kin‐pairs

Using *CKMRsim*, we identified three full‐sibling pairs and 16 HSPs among the 662 genotyped individuals. When comparing the three kin‐finding approaches, *CKMRsim*, *ML‐relate*, and *related*, the results were largely consistent. However, *ML‐relate* was less conservative, identifying eight additional HSPs. On assessing the relatedness values (*r*) among these additional pairs of samples, we noticed the additional related individuals from *ML‐relate* generally had lower *r* values than the rest (Table S3). By including tests for first‐cousins in *CKMRsim*, we identified 196 pairs of individuals that were likely to be distant relatives (e.g. third‐order relatives such as cousins). As *ML‐relate* does not test for third‐order relatives, it may have falsely identified a number of third‐order relatives as half‐siblings. A conservative approach was adopted, whereby the three full‐sibling and 16 HSPs identified using *CKMRsim* were retained for downstream analysis.

Of the 19 sibling‐pairs identified, in four cases the pair of individuals were captured in the same haul. The other sibling pairs were captured between two and 94 km apart (Figure 1, Table 2). Two individuals were each involved in multiple sibling pairs. The majority of sibling pairs involved at least one individual captured just west of the Isles of Scilly (Figure 1). These results suggest a pattern of site fidelity that might violate the assumptions of CKMR, in that they could lead to spatially biased sampling (Conn et al., 2020). Indeed, comparing the geographic distance between half‐siblings with that between all potential pairs suggested that half‐siblings were more likely to be found in closer proximity than expected by chance (Kolmogorov–Smirnov test, *D* = 0.40, *p* = 0.01; Figure S5). However, given that our sampling protocol was systematic rather than opportunistic, and covered an area of the Celtic Sea believed to capture most of the range of the population, we considered any spatial sampling bias to be negligible (Conn et al., 2020).

**TABLE 2:** Sampling and biological details of three full‐sibling (FS) and 16 half‐sibling (HS) pairs of *Dipturus batis* sampled in the Celtic Sea

ID1	Year	Lat, long (decimal)	Sex	Length (cm)	ID2	Year	Lat, long (decimal)	Sex	Length (cm)	Kinship	Distance apart (km)
elasm_1232	2015	49.32 N 6.73 W	M	121	elasm_1401	2017	49.20 N 7.82 W	F	122	FS	80
elasm_1415	2017	49.35 N 7.55 W	F	142	elasm_608	2011	49.97 N 6.85 W	M	132	FS	85
elasm_1378	2017	49.09 N 7.93 W	F	94	elasm_1388	2017	49.27 N 7.67 W	F	84	FS	27
elasm_1351	2017	50.23 N 7.01 W	M	97	elasm_1354	2017	50.23 N 7.01 W	M	115	HS	0
elasm_1078	2015	49.50 N 6.60 W	F	136	elasm_749	2011	50.03 N 6.92 W	F	141	HS	63
elasm_1315	2017	50.11 N 6.80 W	M	120	**elasm_918**	2014	49.95 N 6.83 W	M	117	HS	17
elasm_1338	2017	50.18 N 6.98 W	M	113	elasm_1339	2017	50.18 N 6.98 W	M	129	HS	0
elasm_1234	2015	49.32 N 6.73 W	M	74	elasm_1291	2017	49.73 N 7.22 W	F	78	HS	57
elasm_612	2011	49.97 N 6.85 W	F	127	**elasm_918**	2014	49.95 N 6.83 W	M	117	HS	2
**elasm_744**	2011	50.03 N 6.92 W	M	107	elasm_747	2011	50.03 N 6.92 W	M	123	HS	0
elasm_645	2011	49.97 N 6.85 W	F	122	elasm_956	2014	49.83 N 7.02 W	M	127	HS	19
elasm_884	2014	49.89 N 6.97 W	M	125	elasm_950	2014	49.83 N 7.02 W	F	123	HS	8
elasm_1309	2017	50.11 N 6.80 W	F	134	elasm_685	2011	49.45 N 7.40 W	M	121	HS	85
elasm_1245	2015	49.28 N 6.68 W	M	133	elasm_623	2011	49.97 N 6.85 W	F	123	HS	77
elasm_1311	2017	50.11 N 6.80 W	F	136	elasm_618	2011	49.97 N 6.85 W	F	139	HS	16
elasm_1277	2015	49.53 N 6.38 W	M	119	elasm_1357	2017	50.18 N 6.82 W	F	125	HS	78
elasm_1422	2017	49.48 N 7.44 W	M	125	**elasm_744**	2011	50.03 N 6.92 W	M	107	HS	72
elasm_1242	2015	49.28 N 6.68 W	F	105	elasm_1327	2017	50.11 N 7.02 W	F	127	HS	94
elasm_615	2011	49.97 N 6.85 W	F	132	elasm_651	2011	49.97 N 6.85 W	M	116	HS	0

*Note*: Samples involved in multiple sibling pairs are in bold.

### 3.2 Close‐kin mark‐recapture

We identified 16 HSPs that could be used for the CKMR model, which was fewer than the 21 we expected based on the results of a pilot study (Appendix S1). The age of the samples, estimated using the von Bertalanffy growth parameters proposed by Barreau et al. (2016), ranged from 2 to 27 years of age. The equation generated significant uncertainty when estimating the age of larger individuals, whereas the majority of samples (*N* = 656) were estimated at between two and 15 years of age, the six largest individuals (length 142–148 cm) were estimated at between 16 and 27 years of age. The oldest skate involved in a HSP was estimated at 15 years of age. Therefore, in order to minimize bias and imprecision in our CKMR model while retaining as much pairwise relatedness information as possible, we excluded the six individuals older than 15 years from our analyses. One HSP involved two individuals from the same cohort (i.e. same estimated birth year). Because same‐cohort pairs violate the assumption of independent samples (Bravington, Skaug, et al., 2016), we excluded this HSP and other unrelated same‐cohort pairs from the analysis. The final analysis involved 197,466 pairwise comparisons, down from the original 218,791, of which 15 were HSPs. The cohorts (and hence the modelled years) spanned 1996 $\left(N_{t=0}\right)$ to 2015 ($N_{t=19}$).

**FIGURE 2:**
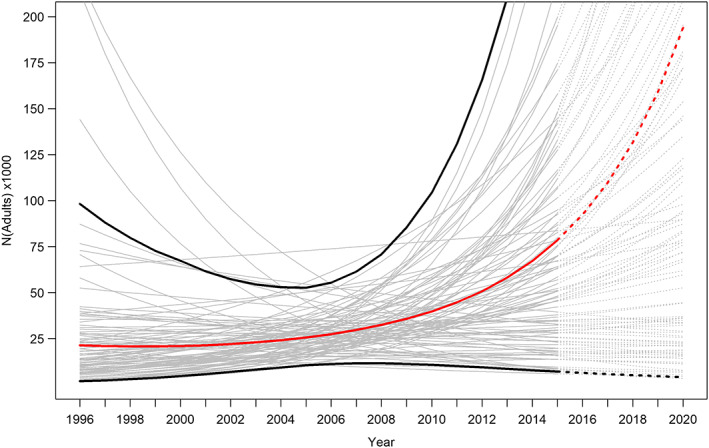
Mean (red line) and 95% credible intervals (black lines) adult breeding abundance of *Dipturus batis* in the Celtic Sea, estimated using CKMR in a Bayesian MCMC framework. 100 random iterations from the model are shown (grey lines). Estimates for the modelled cohorts (solid lines) and years following the last cohort in the model (dotted lines) are shown. Note that the mean tends upwards towards the beginning and end of the time‐series, which is an artefact occurring from taking averages.

As a result of finding relatively few HSPs, the estimates involved fairly large credible intervals (Figure 2, Table S4). The nature of CKMR models is such that abundance estimates have the largest uncertainty at the beginning and end of the time series; the highest precision in our model occurred in the year 2005 $\left(N_{t=10}=25,582;95\%\mathrm{CI}=10,484\text{–}52,644\right)$. The high uncertainty in growth rate estimate precluded us from drawing conclusions on the population trend, though the mean estimate suggested an increasing population trend $\left(r=0.071;95\%\mathrm{CI}=-0.119\;\text{to}\;0.259\right)$. The annual adult survival rate (φ) was estimated at 0.828 (95%CI=0.637–0.984). We underscore that, due to the preliminary age‐at‐length key for the species, age uncertainty could not be meaningfully incorporated into the model, so the uncertainty bounds were likely underestimated. Testing for the sensitivity of the model to erroneous age estimates, we found that re‐assigning ages to individuals had a negligible effect on parameter estimates, but slightly widened the uncertainty bounds (Figure S4).

### 3.3 Genetic diversity and effective population size

Genetic diversity was stable across all sampling years (2011–2017), with the population of *D. batis* maintaining relatively constant levels of observed heterozygosity of ~0.31 across years (Table 3). Overall effective population size (*N*_*e*_) was estimated at 14,832 individuals (95% CI: 11,987–19,416, Table 3). *N*_*e*_ estimates varied across sampling years, with an increase from approximately 14,000 individuals in 2011 to 28,000 individuals in 2015, before a drop to 8500 in 2017. However, confidence intervals were much larger for yearly *N*_*e*_ estimates.

**TABLE 3:** Mean genomic summary statistics for *Dipturus batis* for each sampling year, and overall across years

Sampling year	*N*	*H*_*o*_	*H*_*e*_	*F*	*N*_*e*_
		(*SE*)	(*SE*)	(*SE*)	(95% CI)
2011	158	0.311 (0.002)	0.312 (0.002)	0.004 (0.001)	13,752 (7930–51,118)
2014	155	0.301 (0.002)	0.311 (0.002)	0.031 (0.001)	19,663 (8642–∞)
2015	204	0.309 (0.002)	0.311 (0.002)	0.007 (0.001)	28,191 (19,981–47,793)
2017	145	0.315 (0.002)	0.313 (0.002)	−0.005 (0.001)	8489 (4467–79,490)
Overall	662	0.309 (0.001)	0.312 (0.001)	0.009 (0.001)	14,832 (11,987–19,416)

*Note*: Sample sizes (*N*), observed heterozygosity (*H*_*o*_), expected heterozygosity (*H*_*e*_), and fixation index (*F*) are shown together with their standard error, calculated in GenAlEx (v 6.5, Peakall & Smouse, 2006, 2012). Effective population sizes (*N*_*e*_) and their 95% confidence intervals are also shown, calculated using the linkage‐disequilibrium method in NeEstimator (v 2.1, Do et al., 2014) at a critical value of 0.05.

### 3.4 Catch per unit effort

**FIGURE 3:**
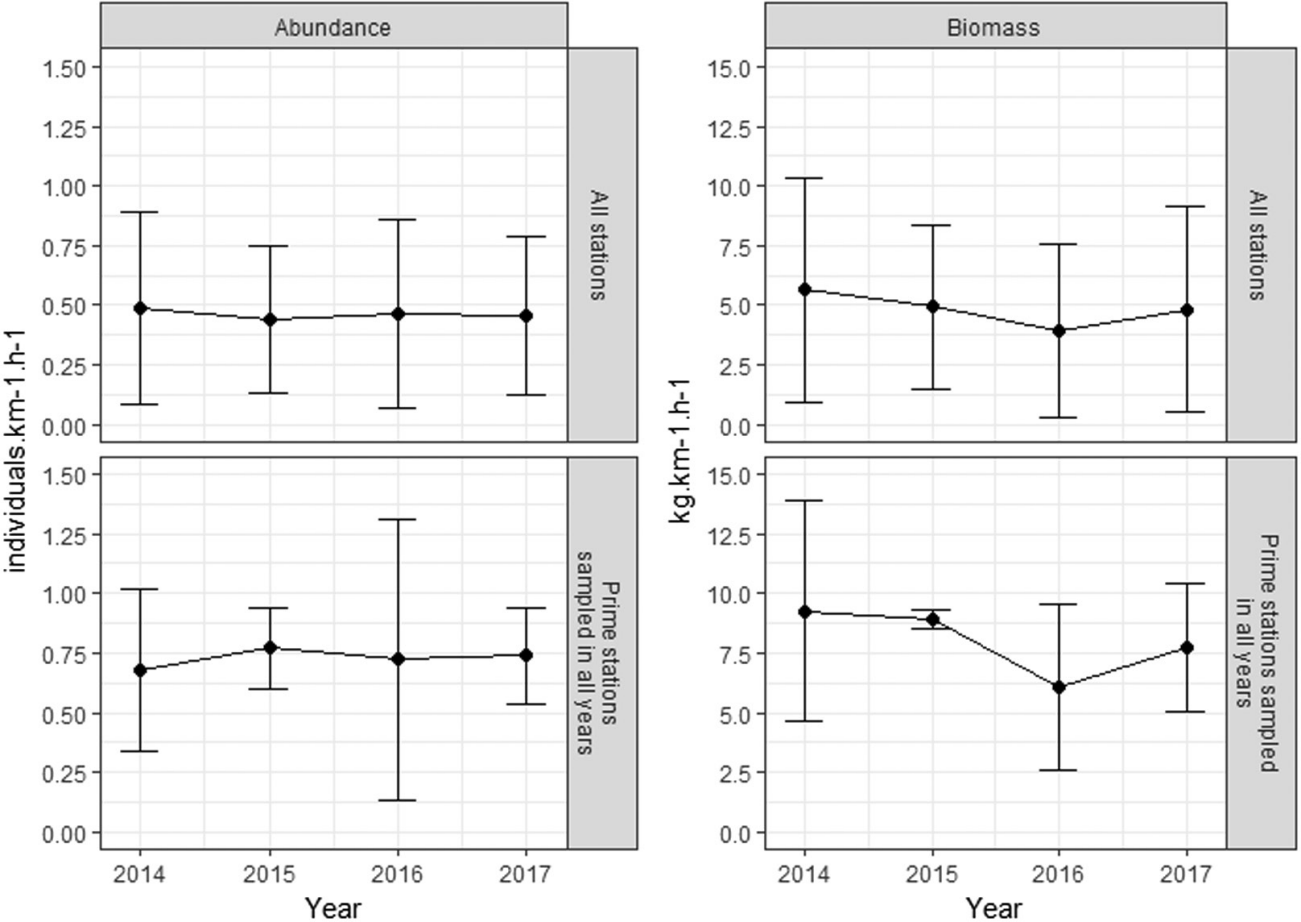
Temporal changes in CPUE of *Dipturus batis* (left panel: Abundance; right panel: Biomass) for all stations fished (top) and for four stations sampled each year (bottom) during fishery‐dependent common skate surveys in the Celtic Sea by CEFAS in collaboration with fishing industry from 2014 to 2017.

Across the 2014–2017 survey period, the mean CPUE by abundance and biomass for *D. batis* remained relatively stable. Mean CPUE ranged from 0.44–0.49 individuals km^−1^ h^−1^ and, in terms of biomass, from 3.96–5.66 kg km^−1^ h^−1^, with notable standard deviations of the mean (Figure 3).

Only four stations were fished in all four survey years (stations C03, C04, C07 and C09 in Bendall et al., 2018), and the mean CPUE at these sites ranged from 0.68 to 0.77 individuals km^−1^ h^−1^ and 6.09–9.27 kg km^−1^ h^−1^ (Figure 3). The lowest recorded mean CPUE (biomass) was recorded in 2016, at 3.96 kg km^−1^ h^−1^ for all stations fished, and 6.09 for kg km^−1^ h^−1^ for the four stations that were fished each year.

## 4 DISCUSSION

In this study, we evaluated the suitability of CKMR as a demographic modelling tool for a data‐deficient and critically endangered benthic elasmobranch population, the Celtic Sea population of blue skate *D. batis*. Using samples collected during fishery‐dependent common skate surveys from 2011 to 2017, we implemented a HSP CKMR model to generate the first estimates of adult breeding abundance, adult population growth rate, and adult survival rates for the population. In addition, the spatio‐temporal distribution of sibling pairs supported the results of Bendall et al. (2018) suggesting that *D. batis* exhibit site fidelity and that an area of critical habitat may occur near the Isles of Scilly. Despite limitations owing to the limited number of kin pairs identified and the data‐deficient nature of *D. batis*, CKMR represents a promising demographic modelling tool for the species, as demonstrated by comparing results from it with molecular estimates of effective population size (*N*_*e*_), genetic diversity and CPUE, the last a more familiar estimate in fisheries science. We discuss the limitations of these estimates, and evaluate the potential of CKMR as a tool to estimate contemporary population demographic parameters, and so a population's evolutionary potential, for this and other data‐deficient elasmobranchs.

The results support anecdotal information that *D. batis* is locally abundant in the Celtic Sea. CPUE estimates remained consistent both in terms of biomass and abundance, indicating a stable population in the short term from 2014 to 2017; estimates of genetic diversity also remained stable across sampling years. The CKMR results suggested that this level of relative abundance corresponds to an adult breeding population in the order of N≈25,000 individuals. However, the CKMR estimates should be interpreted with caution since they were based on the inclusion of relatively few (15) HSPs, and additional parameters such as age uncertainty could not be incorporated due to the preliminary nature of life‐history data on the species. The only other known estimates of abundance for the population come from Barreau et al. (2016)’s estimates of CPUE, which were based on fishery‐independent bottom trawl data. These authors reported a moderate increase in CPUE from 2009 to 2014. Altogether, the results from both molecular and CPUE approaches seem to suggest a stable, possibly increasing, population of *D. batis* in the Celtic Sea following the 2009 landing ban, at least until 2017.

Estimates of *N*_*e*_ were in the order of ~15,000 individuals, which is above the generally accepted conservation thresholds (e.g. of 500 or 5000, see Allendorf et al., 2013) and suggests that the Celtic Sea population of *D. batis* is at a relatively low risk of inbreeding depression. We noted a decline in *N*_*e*_ in 2017; however, our *N*_*e*_ estimates should be interpreted with caution: estimates were derived from a mixed‐age group of individuals and therefore do not reflect *N*_*e*_ per generation, which could have been assessed by estimating *N*_*e*_ for each cohort should sample sizes have allowed it (e.g. as in Waples et al., 2018), and the high‐throughput sequencing data from which they were derived may be subject to bias due to violations of the assumption of unlinked loci (Waples et al., 2016).

The CKMR estimates suggested an annual adult survival rate of $\varphi =0.83\;\left(95\%\mathrm{CI}=0.64\text{–}0.98\right)$ for *D. batis* in the Celtic Sea. This is higher than the 0.64 estimated for the larger flapper skate (*D. intermedius*) based on the results of a mark‐recapture programme on the west coast of Scotland (Neat et al., 2015), although their estimate included juveniles which likely experience higher natural mortality rates than adults on account of their smaller size. The higher mortality of *D. intermedius* might also reflect a greater susceptibility to fishing mortality for this species (Brander, 1981). In comparison with existing elasmobranch CKMR studies, the adult survival rate estimates for *D. batis* were lower than for adult white shark *Carcharodon carcharias* (Hillary et al., 2018) and grey nurse shark *Carcharias taurus* (Bradford et al., 2018), both estimated at over 0.90. Such a high survival rate might be expected for large oceanic predators given their higher trophic position. It is worth noting that mortality rates for *D. batis* discards (accidental catches) in commercial tangle‐net and trawl fisheries have been estimated at 38.5% (Ellis et al., 2016) and 33.5% (Barreau et al., 2016), respectively, while skates in general experience mortality rates as high as 45% following capture in UK trawl fisheries (Enever et al., 2009). The potential rates of mortality that can be inflicted on skate populations by commercial fisheries may therefore be consequential, supporting the need for rigorous monitoring and implementation of targeted conservation actions.

There is growing evidence of philopatric, site‐attached (e.g. Feutry et al., 2017; Neat et al., 2015), and aggregating behaviour (Lieber et al., 2020; Thorburn et al., 2018) exhibited by elasmobranchs, which may relate to preferential feeding, reproductive or nursery grounds that could qualify as conservation areas. The full‐ and half‐sibling pairs identified in this study were sampled in closer proximity to one another (<100 km apart) than would be expected by chance, even when collected several years apart, indicative of site fidelity. Of particular interest was the high density of sibling pairs that occurred in the waters west of the Isles of Scilly. This location corresponded to an area thought to be ‘biologically important’ by Bendall et al. (2018) where there were increased catches of immature and mature males and females, and the occurrence of sexually active males and egg‐bearing females had been observed. Our results therefore support earlier observations, and identify a site warranting further investigation, one that could perhaps benefit from targeted conservation actions.

This study allows us to evaluate sampling‐design considerations for improved implementations of CKMR on *D. batis* and other elasmobranchs sharing similar life‐history characteristics. We could establish that the sampling effort required for a large‐bodied skate might conceptually lie between that of a ‘teleost fish’ (e.g. Bravington, Grewe, et al. (2016) identified 45 parent‐offspring pairs among 14,000 genotyped southern bluefin tuna, estimating an adult abundance of ~2 million individuals) and a ‘shark’ (e.g. Hillary et al. (2018) identified 21 HSPs among 100 genotyped white sharks, estimating an adult abundance of 280–650 individuals). Though we effectively maximized the genotyped sample size in our study based on cost, DNA quality and population dynamic considerations, we estimate that in order to obtain CKMR estimates at the precision required of fisheries stock assessments (i.e. corresponding to at least ~45 kin pairs, according to Bravington, Skaug, et al., 2016), sampling should continue for the Celtic Sea *D. batis* population until ~1100 individuals have been genotyped, based on the assumption that the number of kin‐pairs identified increases quadratically with sample size (Bravington, Grewe, et al., 2016; Appendix S1).

Furthermore, the site fidelity of *D. batis* suggests that sampling effort should be increased longitudinally and across several stations in order to avoid sampling litter mates (i.e. siblings born in the same year), which complicate CKMR as they violate the assumption of independent samples (Bravington, Skaug, et al., 2016). In practice, sampling litter mates that are the offspring of an adult with especially high reproductive success in a particular year may downwardly bias the abundance estimate; site fidelity would also increase the probability of sampling a litter mate, especially if these two siblings happen to share a trait conferring increased survival, and therefore capture. This is why we opted for a conservative approach by omitting same‐cohort pairs. In contrast, it may be argued that given the level of uncertainty in our age estimates, the same‐cohort pairs could have been retained.

Despite the preliminary nature of the age‐at‐length key used in this study (Barreau et al., 2016), it provides the best available age estimator for *D. batis* at the time of writing. Accurate age estimation remains a challenge in elasmobranch population assessment, often requiring lethal sampling to obtain vertebral growth readings. However, novel statistical approaches now provide an opportunity to model age based on individual size, though these require considerable sampling efforts (Régnier et al., 2021).

The variable catchability of elasmobranchs by different sampling gears is also an issue to consider. The trammel‐net surveys captured mostly sub‐adults and young adults (length range = 66–148 cm; Table 1). The relative lack of small juveniles may have limited the number of cohorts sampled and might explain the absence of parent‐offspring pairs in our samples, which would have enabled total adult abundance estimation using a parent‐offspring pair CKMR approach (Bravington, Grewe, et al., 2016; Bravington, Skaug, et al., 2016). In contrast, the bottom trawl data used in the CPUE estimates of Barreau et al. (2016) contained a wide range of size classes (20–140 cm), including an abundance of juveniles. These patterns suggest that alternative sampling methods such as trawling may yield a wider size (and age) range of skates that could be advantageous for CKMR abundance estimation, although they may be more destructive.

In summary, our CKMR and *N*_*e*_ estimates provide a first approximation of the number of breeding adult *D. batis* in the Celtic Sea, complementing CPUE estimates, but they would benefit from further validation by other methods. At present, experimental validation of CKMR has been limited to parent‐offspring pair models in riverine brook trout (*Salvelinus fontinalis*) populations using mark‐recapture methods (Marcy‐Quay et al., 2020; Ruzzante et al., 2019). The long‐term tagging of *D. batis* in the Celtic Sea, initiated by Bendall et al. (2018) and Barreau et al. (2016), may provide a useful dataset to validate our CKMR estimates once a sufficient number of individuals are recaptured. Thereafter, more precise demographic models could be developed and applied to the population, and to other elasmobranchs sharing similar life‐history traits as *D. batis*.

Despite the limitations discussed, CKMR has proven to be a relatively cost‐effective method of estimating important population demographic parameters for a critically endangered data‐deficient elasmobranch; the increasing affordability of sequencing technologies is making it possible to genotype large numbers of samples from single sampling events, while offsetting the increasing costs of the repeated surveys at sea needed to physically recapture individuals (which would also incur additional stress on the animals). In addition to the results of a recent seascape genomics study (Delaval et al., 2022), the present findings improve the status of knowledge for what is likely one of the few remaining large populations of *D. batis*. Together, these studies demonstrate the additive value of genomic SNP‐based approaches to elasmobranch research, which can be implemented in different frameworks (e.g. evolutionary, demographic, and environmental) to address longstanding questions of immediate relevance to conservation.

## CONFLICT OF INTEREST

The authors declare no conflicts of interest.

## Supporting information


Appendix S1
Appendix S2
Appendix S3
Appendix S4
Appendix S5

## Data Availability

The data that support the findings of this study are openly available in the Dryad Digital Repository https://doi.org/10.5061/dryad.n2z34tn0g.
