# Supplementary material for: Evaluating the suitability of close‐kin mark‐recapture as a demographic modelling tool for a critically endangered elasmobranch population
Source: Evol Appl. 2022 Sep 3;16(2):461–73. doi: 10.1111/eva.13474 (PMC9923483; doi:10.1111/eva.13474)
Supplement: Supplementary file 1 — Appendix S1 Appendix S2 Appendix S3 Appendix S4 Appendix S5 [file EVA-16-461-s001.docx]

**Supplementary material**

Appendix S1: CKMR pilot study

In order to assess the feasibility of CKMR for our study system and design an optimal sampling protocol given the samples available, we performed a preliminary analysis on 387 blue skates from the Celtic Sea collected during CEFAS surveys between 2011 and 2017, which were DArTseq^TM^ genotyped in a recent population and seascape genomic study (Delaval et al., 2022). This allowed us to i) establish the level of precision with which the genotyping panel can identify related individuals (i.e. calculate false-positive and false-negative rates), ii) tailor our research questions, and iii) design an optimal sampling strategy to address those questions.

Among the 387 skates, we identified one full-sibling pair and seven half-sibling pairs. False-positive rates in relationship tests were very low (FPR ≤ 5.62x10^-56^ using the R package CKMRsim, unpublished data), suggesting the genotyping panel identifies kin-pairs with high confidence. Note that the ~6,000 loci were considered as independent in this preliminary analysis, using the package’s default importance sampling approach. Kin-pairs were found in close proximity to one another, and there was limited gene flow with neighbouring populations (Delaval et al., 2022). Therefore, for the purposes of the CKMR model, we assumed the Celtic Sea population to be a closed population.

CKMR generally consists of two approaches. The first identifies parent-offspring pairs to estimate total adult abundance, while the second identifies half-sibling pairs to estimate the number of breeding adults (Bravington et al., 2016b). The more related pairs are identified, the higher the precision of the model, and an ideal CKMR study is reportedly one in which the coefficient of variation (CV) is 0.15, which is achieved when a dataset includes at least ~45 kin-pairs (Bravington et al., 2016b). Having primarily identified half-sibling pairs in our dataset, we opted for a half-sibling pair (HSP) CKMR model. Seven HSPs implies a CV of ~0.38, so sampling effort would need to be increased to obtain precise estimates of population size. To achieve a CV of ~0.15 would in theory require genotyping 1,034 individuals (equations shown below).

The maximum number of individuals we could genotype in our study, due to cost and DNA quality considerations, was 683 individuals. With this sampling effort, we might expect to find 21 HSPs. We focused our efforts on the HSP approach by primarily genotyping juveniles and young adults, while including a wide size range of individuals to minimize sampling bias and maximise the number of cohorts covered in our model.

Useful equations:

To approximate the coefficient of variation (CV):

$CV\approx\frac{1}{\sqrt{H}}$

where H is the number of related pairs found in the data.

Number of related pairs expected for a given sample size:

$H_{2}=\frac{H_{1}}{N_{1}\times(N_{1}-1)/2}\times\frac{N_{2}\times(N_{2}-1)}{2}$

Where H_1_ is the number of pairs found initially, H_2_ is the number of pairs expected with a change of sample size, N_1_ is the initial sample size, and N_2_ is the new sample size.

Appendix S2: Summary of filtering steps

Table S1: Summary of SNP filtering steps

| **Filtering step** | **# Loci removed** | **# Loci remaining** |
| --- | --- | --- |
| Raw data |  | 25,131 |
| Call rate < 95% | 7,857 | 17,274 |
| Duplicate loci | 1,081 | 16,193 |
| Monomorphic loci | 3,346 | 12,847 |
| Minor allele frequency (MAF) < 0.05 | 2,744 | 10,103 |
| Loci out of Hardy-Weinberg proportions. | 3,674 | 6,429 |
| Loci in linkage disequilibrium | 138 | 6,291 |


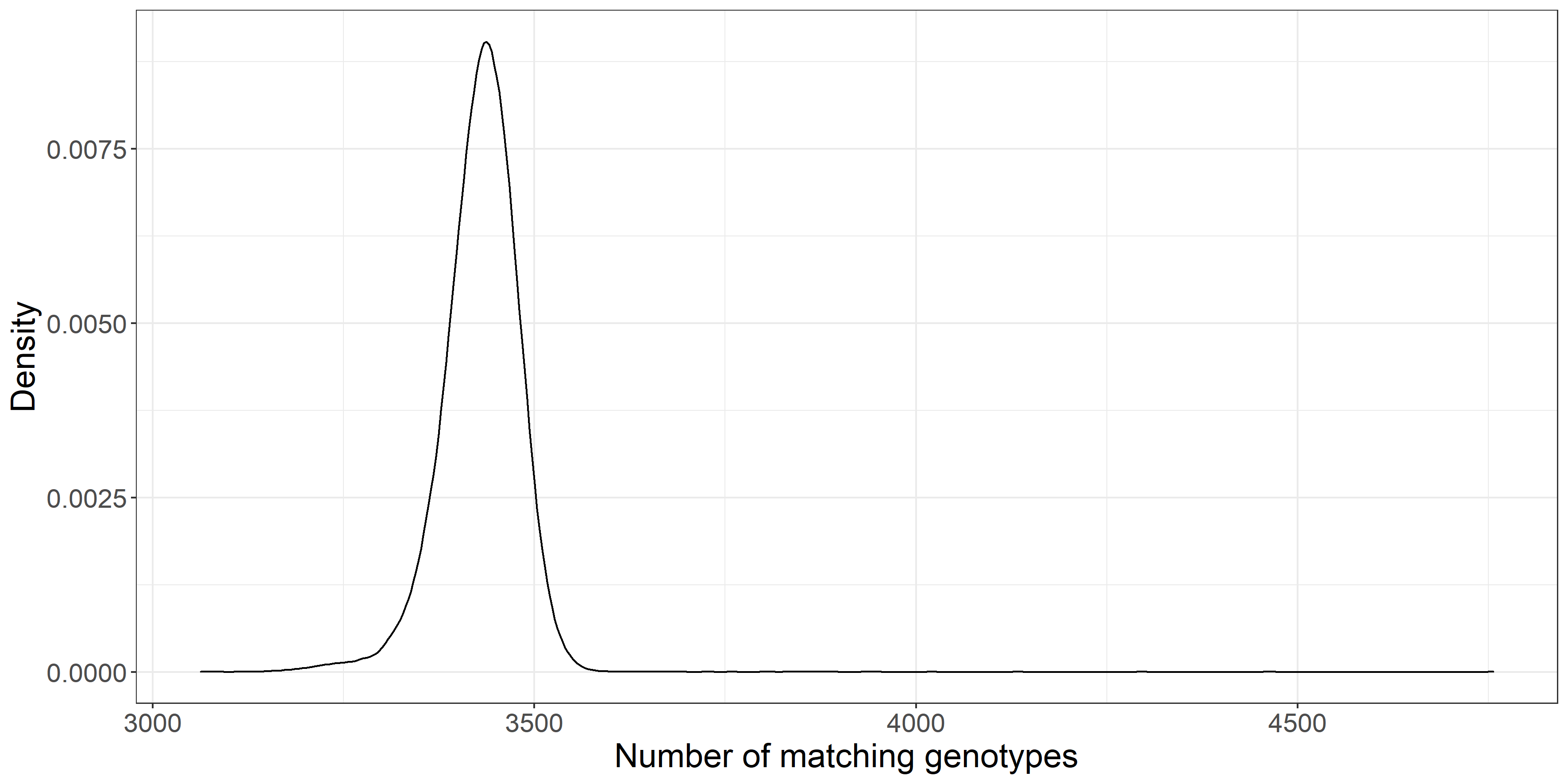


Figure S1: Density distribution of the number of matching genotypes (out of 6,291 loci) across all 218,791 pairwise comparisons of blue skate (involving 662 individuals). The data were used to filter out any duplicate samples, based on a threshold of 621 mismatching (5,662 matching) genotypes.

Appendix S3: Finding related individuals

Using the R package *CKMRsim* (Anderson, [https://doi.org/10.5281/zenodo.3519358](https://doi.org/10.5281/zenodo.820162)), we simulated 5,000 pairs of each of five relationship categories using observed allele frequencies to identify appropriate decision-making thresholds. These were: unrelated (U), first-cousin (FC), half-sibling (HS), full-sibling (FS), and parent-offspring (PO) pairs. To avoid treating all 6,291 loci as independent, since some degree of linkage must be accommodated across a finite genome, we simulated a linkage map in the absence of a reference genome. We distributed the 6,291 loci randomly across a ‘mock’ genome with the following characteristics: genome of size n=49 (based on findings for Rajidae in Stingo and Rocco (2001)) and length L=4.3Gb (a conservative approximation based on the white shark genome, NCBI), and a smallest chromosome of size sl=1/15 (proportion of largest chromosome).

We computed the log-likelihoods for all pairs of individuals across the following tests: FC vs. U, HS vs. FC, FS vs. HS, and PO vs. FS. In this order, we could a) identify which individuals were related versus unrelated, and b) progressively filter pairs of individuals categorically from less related (FC) to most related (PO), based on the defined log-likelihood thresholds in our simulations. Documentation and R code are openly available in the package’s repository.

Reference: Stingo, V. & Rocco, L. (2001). Selachian cytogenetics: a review. *Genetica, 111*, 329-347.


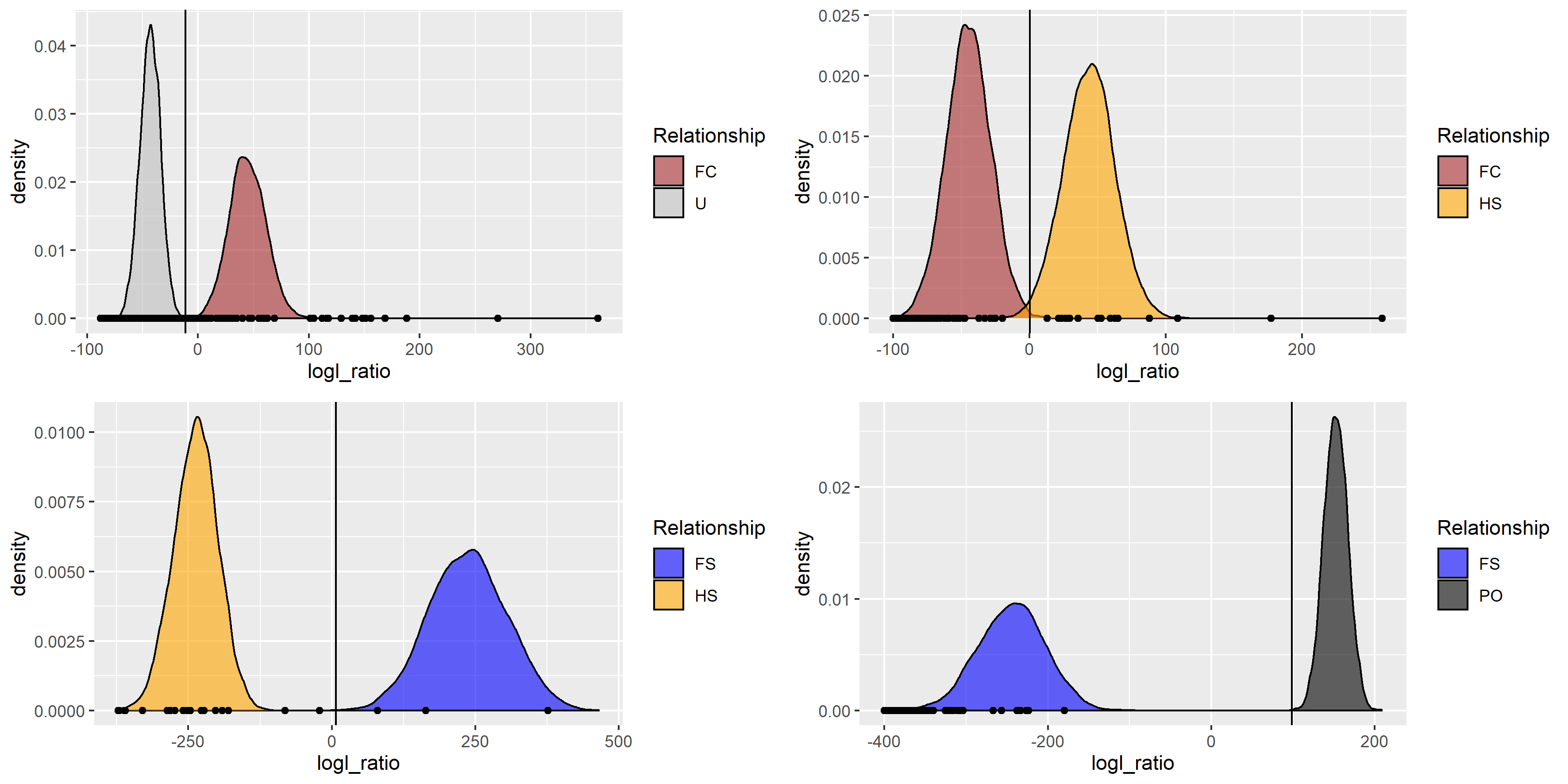


Figure S2: Simulated density distributions in log-likelihood (logl) space for four relationship tests, generated using *CKMRsim*, in order of increasing relatedness from unrelated (U) to first-cousin (FC), half-sibling (HS), full-sibling (FS), and parent-offspring (PO). Points indicate the observed log-likelihood ratio for each pair in our data for each test. Vertical lines represent the decision-making thresholds used (logl cut-offs in Supplementary Table 2).

Table S2: Decision making thresholds in four relationship tests involving unrelated (U), first-cousin (FC), half-sibling (HS), full-sibling (FS) and parent-offspring (PO) pairs, showing false-negative rates (FNR) and their associated with false-positive rates (FPR), log-likelihood ratio (logl) cut-offs, and the number of positive hits for each test. Note that, of the 215 related pairs identified, 16 were half-siblings and three were full-siblings. FPRs smaller than 0.001 could not be computed accurately with the vanilla Monte Carlo method used.

| **Test** | **FNR** | **FPR** | **logl cut-off** | **No. pairs found** |
| --- | --- | --- | --- | --- |
| FC/U | 0.0001 | <0.001 | -11.3 | 215 |
| HS/FC | 0.01 | 0.003 | 0.11 | 19 |
| FS/HS | <1x10^-8^ | <0.001 | 6.82 | 3 |
| PO/FS | <1x10^-8^ | <0.001 | 98.6 | 0 |

Table S3: Summary of full-sibling (FS) and half-sibling (HS) pairs among genotyped *Dipturus batis* from the Celtic Sea, identified using *CKMRsim* and *ML-relate*, and the Wang relatedness score (r) for those pairs calculated using *related*. Eight of the HS identified in *ML-Relate* were identified as first-cousins (FC) in *CKMRsim*.

| **ID1** | **ID2** | ***CKMRsim*** | ***ML-Relate*** | **r (*related*)** |
| --- | --- | --- | --- | --- |
| elasm_1232 | elasm_1401 | FS | FS | 0.5687 |
| elasm_1415 | elasm_608 | FS | FS | 0.4596 |
| elasm_1378 | elasm_1388 | FS | FS | 0.4351 |
| elasm_1351 | elasm_1354 | HS | HS | 0.3716 |
| elasm_1078 | elasm_749 | HS | HS | 0.2533 |
| elasm_1315 | **elasm_918** | HS | HS | 0.2625 |
| elasm_1338 | elasm_1339 | HS | HS | 0.3407 |
| elasm_1234 | elasm_1291 | HS | HS | 0.2495 |
| elasm_612 | **elasm_918** | HS | HS | 0.2454 |
| **elasm_744** | elasm_747 | HS | HS | 0.2359 |
| elasm_645 | elasm_956 | HS | HS | 0.2586 |
| elasm_884 | elasm_950 | HS | HS | 0.2413 |
| elasm_1309 | elasm_685 | HS | HS | 0.2102 |
| elasm_1245 | elasm_623 | HS | HS | 0.2169 |
| elasm_1311 | elasm_618 | HS | HS | 0.2159 |
| elasm_1277 | elasm_1357 | HS | HS | 0.2219 |
| elasm_1422 | **elasm_744** | HS | HS | 0.2104 |
| elasm_1242 | elasm_1327 | HS | HS | 0.2342 |
| elasm_615 | elasm_651 | HS | HS | 0.2025 |
| elasm_1224 | elasm_1311 | FC | HS | 0.1553 |
| elasm_1245 | elasm_746 | FC | HS | 0.1490 |
| elasm_1367 | elasm_758 | FC | HS | 0.1519 |
| elasm_1245 | elasm_1390 | FC | HS | 0.1553 |
| elasm_652 | elasm_653 | FC | HS | 0.1634 |
| elasm_1143 | elasm_621 | FC | HS | 0.1907 |
| elasm_1322 | elasm_1339 | FC | HS | 0.1907 |
| elasm_718 | elasm_986 | FC | HS | 0.1441 |

Appendix S4: Bayesian Markov-Chain Monte-Carlo (MCMC) model parameters and outputs

Starting parameters:

$N_{t=0}=\ln\left( 10,000 \right)$ $r=0.01$ $\varphi=0.90$

Probability density functions (PDFs) used for each parameter:

$N_{t=0}:Normal PDF, where \mu=\ln\left( 20,000 \right)and \sigma=ln(10,000)$

$r:Uniform PDF [-1,1]$

$\varphi:Uniform PDF [0,1]$

MCMC inference proceeded with a Metropolis-Hastings sampler where new parameter candidates were drawn from a uniform distribution centred about the previous iteration’s value. The support for each uniform distribution had the following ranges:

$\mathrm{For}N_{t=0}:\ln(2000)$

$\mathrm{For}r:0.4$

$\mathrm{For}\varphi:0.4$

MCMC parameters:

Number of iterations = 1 million

Sampling frequency = every 100 runs

Burn-in = 100,000

Table S4: Results of the Bayesian analysis, showing parameter estimates and credible intervals. The initial model year ($N_{t=0}$) refers to the year 1996.

| Parameter | Estimate | Lower 95% CI | Upper 95% CI |
| --- | --- | --- | --- |
| ln($N_{t=0}$) | 9.427 | 7.486 | 11.495 |
| *r* | 0.071 | -0.119 | 0.259 |
| $\varphi$ | 0.828 | 0.637 | 0.984 |


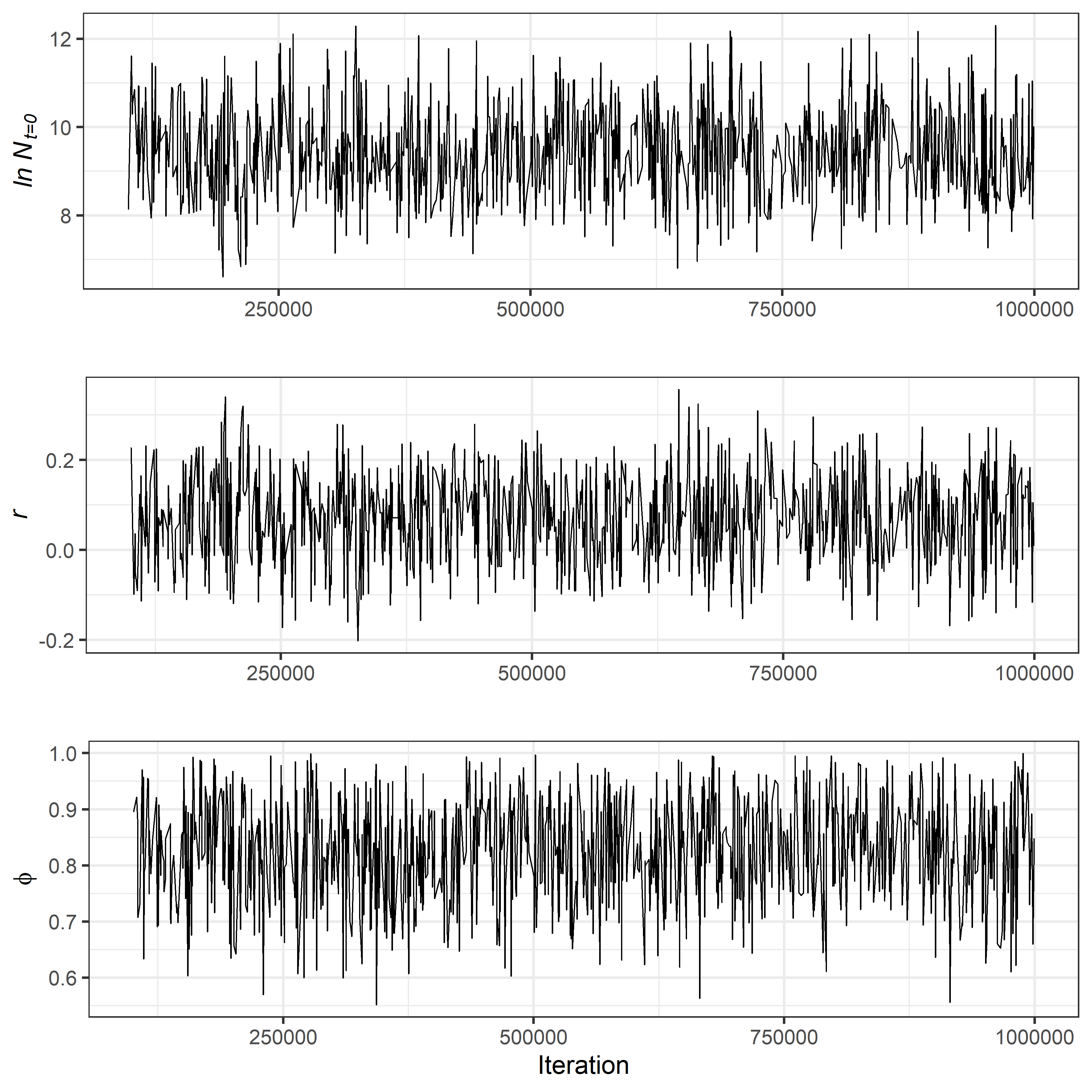


Figure S3: A random subset of 1000 points visited (out of 1 million) by the MCMC chain, for each of the estimated parameters: initial adult breeding abundance ($N_{t=0}$ expressed in natural log), population growth rate (*r*), and survival rate ($\varphi$).

Appendix S5: CKMR age-sensitivity analysis

To assess the sensitivity of the CKMR model to erroneous age estimates, we performed 100 CKMR iterations in a maximum-likelihood framework (to save on computation time), using the *optim* function in R, to maximise the pairwise log-likelihood of the model across the parameter space of the three variables, $N_{t=0}, r$ and $\varphi$ (as in *Equation 4*, but excluding the priors). We re-assigned ages to individuals for each iteration. Lacking a validated age-at-length model, we randomly adjusted the ages of individuals <10 years old within a range of -2 to 2 years, and that of individuals ≥10 years old within a range of -4 to 4 years, thereby reflecting the fact that age uncertainty increases with size. These bounds are slightly narrower than those depicted for *D. intermedius*, *D. batis*’ larger congener, in Regnier et al. (2021)’s Figure 5.

Reference: Régnier, T., Dodd, J., Benjamins, S., Gibb, F. M., & Wright, P. J. (2021). Age and growth of the Critically Endangered flapper skate, *Dipturus intermedius*. *Aquatic Conservation: Marine and Freshwater Ecosystems*, *May*, 1–8. https://doi.org/10.1002/aqc.3654


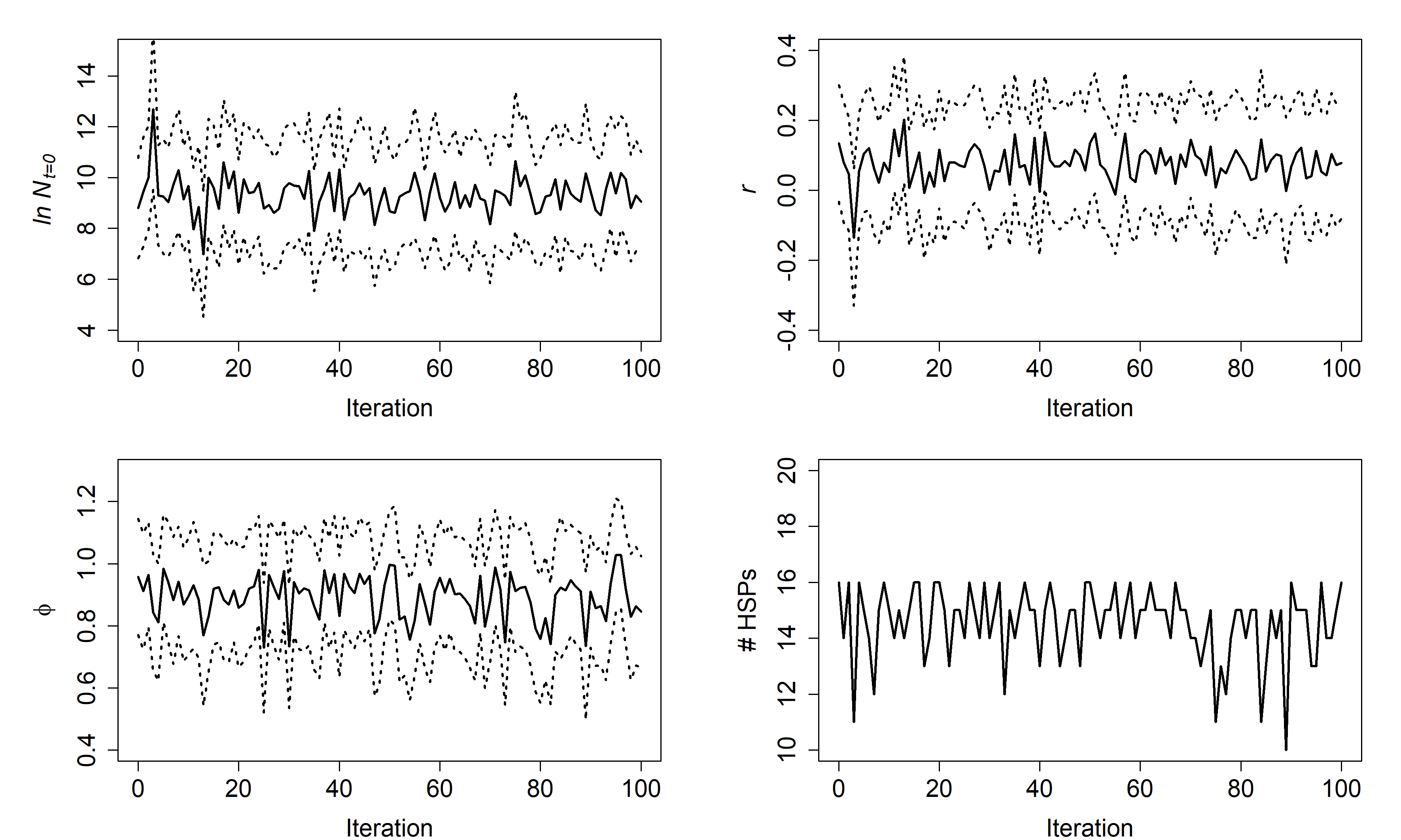


Figure S4: Parameter estimates (solid line) and 95% confidence intervals (dotted lines) of initial adult breeding abundance ($N_{t=0}$ expressed in natural log), population growth rate (*r*), and survival rate ($\varphi$) for 100 iterations of CKMR using maximum-likelihood. For each iteration, ages of individuals were re-sampled, thereby altering the number of retained half-sibling pairs (HSPs), since the CKMR model excludes same-cohort comparisons. Note that estimates of $\varphi$ sometimes exceed 1, since no bounds were set in the maximum-likelihood framework.

Appendix S6: Testing for spatial mixing of samples


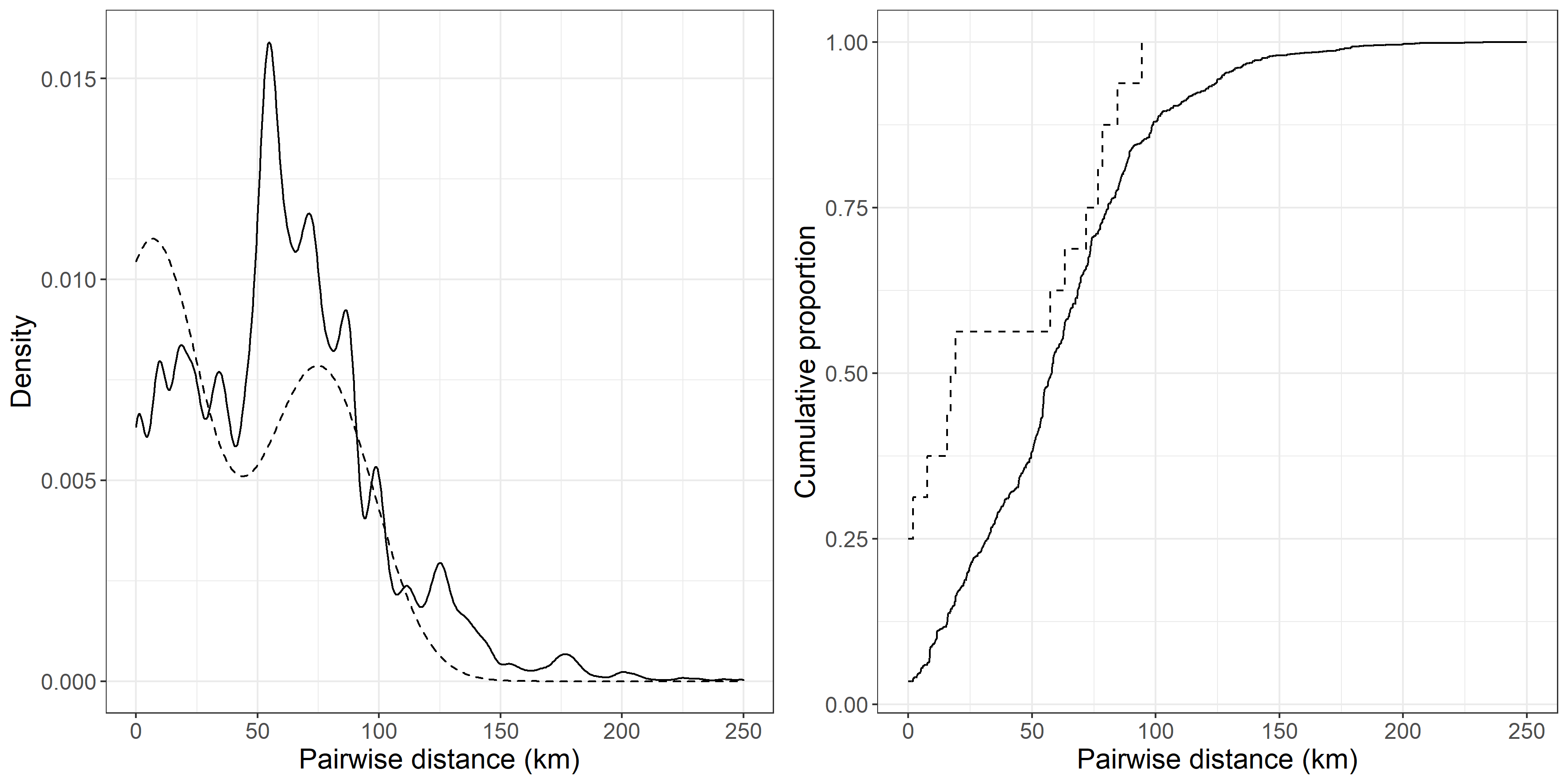


Figure S5: Density distributions (left) and cumulative proportions (right) of geographic straight-line distances between half-sibling pairs (dashed line, n=16) and all potential pairs (solid line, n=218,791) of blue skate in the Celtic Sea. A two-sample Kolmogorov-Smirnov test suggests a significant difference between the two cumulative proportion curves (D = 0.40, p = 0.01), i.e. half-sibling pairs are found in closer proximity than expected by chance.
